# [^18^F]tetrafluoroborate-PET/CT enables sensitive tumor and metastasis *in vivo* imaging in a sodium iodide symporter-expressing tumor model

**DOI:** 10.1038/s41598-017-01044-4

**Published:** 2017-04-19

**Authors:** S. Diocou, A. Volpe, M. Jauregui-Osoro, M. Boudjemeline, K. Chuamsaamarkkee, F. Man, P. J. Blower, T. Ng, G. E. D. Mullen, G. O. Fruhwirth

**Affiliations:** 1 King’s College London, Imaging Chemistry and Biology, Division of Imaging Sciences and Biomedical Engineering, 4th Floor Lambeth Wing, St. Thomas’ Hospital, London, SE1 7EH UK; 2 King’s College London, The Richard Dimbleby Department of Cancer Research, Randall Division of Molecular Biophysics and Cancer Division, Guy’s Campus, London, SE1 1UL UK; 3 UCL, Cancer Institute, Paul O’Gorman Building, London, WC1E 6BT UK

## Abstract

Cancer cell metastasis is responsible for most cancer deaths. Non-invasive *in vivo* cancer cell tracking in spontaneously metastasizing tumor models still poses a challenge requiring highest sensitivity and excellent contrast. The goal of this study was to evaluate if the recently introduced PET radiotracer [^18^F]tetrafluoroborate ([^18^F]BF_4_^−^) is useful for sensitive and specific metastasis detection in an orthotopic xenograft breast cancer model expressing the human sodium iodide symporter (NIS) as a reporter. *In vivo* imaging was complemented by *ex vivo* fluorescence microscopy and γ-counting of harvested tissues. Radionuclide imaging with [^18^F]BF_4_^−^ (PET/CT) was compared to the conventional tracer [^123^I]iodide (sequential SPECT/CT). We found that [^18^F]BF_4_^−^ was superior due to better pharmacokinetics, *i.e*. faster tumor uptake and faster and more complete clearance from circulation. [^18^F]BF_4_^−^-PET was also highly specific as in all detected tissues cancer cell presence was confirmed microscopically. Undetected comparable tissues were similarly found to be free of metastasis. Metastasis detection by routine metabolic imaging with [^18^F]FDG-PET failed due to low standard uptake values and low contrast caused by adjacent metabolically active organs in this model. [^18^F]BF_4_^−^-PET combined with NIS expressing disease models is particularly useful whenever preclinical *in vivo* cell tracking is of interest.

## Introduction

Most cancer-related deaths are a result of metastatic disease^1^. Reliable imaging of cancer metastases in model systems is difficult to achieve and may require a multi-modal imaging approach. *In vivo* cell-tracking methods can be used as a tool to preclinically monitor the presence, distribution, quantity, and viability of malignant cells in primary tumors and distant metastases, noninvasively, longitudinally and in real time in the body. Small animal radionuclide imaging techniques such as positron emission tomography (PET) and single photon emission computed tomography (SPECT) offer good depth penetration and the possibility of absolute quantification^2^ with resolution of <1 mm^3, 4^. Bioluminescence imaging is less expensive but is affected by differential light absorption and poor depth penetration, precluding full 3D quantification^5^. Fluorescence whole-body imaging is less sensitive as compared to bioluminescence technology^5^, but has the advantage of enabling fluorescence microscopy-afforded histology. Therefore, radionuclide and fluorescence imaging complement one another, ranging from the whole-body level to the (sub-)cellular scale. PET and SPECT are well-suited for tracking cells in preclinical models, as the imaging agents are versatile and can be introduced conveniently through direct cell labeling, tracer uptake by endogenous transport proteins, tracer binding through specific high-affinity interactions with surface molecules, or by using reporter gene strategies.

The sodium/iodide symporter (NIS) has a long imaging history because there is a variety of γ-camera/SPECT radiotracers including [^123^I]iodide, [^125^I]iodide and ^99m^TcO_4_^−^, all of which are widely available at comparatively low cost without the need for specialist radiochemistry facilities. NIS has been the basis of molecular imaging and radionuclide therapy of thyroid disease for many years^6, 7^. It is naturally highly expressed in the thyroid and, at lower levels, in only a limited number of extra-thyroidal tissues (*i.e*. salivary, lachrymal and lactating mammary glands, and the stomach), thereby enabling good contrast imaging in other body regions. As a symporter, its anion import is coupled to sodium import, which is fuelled by the electrochemical gradient of sodium ions across cell membranes^7^. Therefore, NIS reports on viable cells only. NIS is highly homologous between human, rat and mouse^7^ and has so far not been reported to exert any toxicity upon ectopic expression in non-thyroidal cells. It has also not been reported to trigger host immune responses (in human, mouse and rat), which can be a concern when foreign reporters are used in the context of fully immunocompetent animals. The combination of ectopic NIS expression with NIS radiotracers has been used to assess promoter activity^8–10^, gene expression^11–16^ and most recently to monitor cells in therapeutic settings ([^131^I]iodide and ^188^ReO_4_^−^)^17–20^.

The quest to image ever smaller cell populations *in vivo*, for example in gene and cell therapy but also cancer metastases research, renders *in vivo* imaging sensitivity and resolution in such whole-body cell tracking applications paramount. Generally, PET has been considered to be superior to SPECT in this respect, particularly in large animals and humans. Accordingly, the PET isotope [^124^I]iodide has been introduced for PET imaging in thyroid disease and NIS reporter gene imaging^21^. However, ^124^I undergoes a complex decay scheme^22^ with a low abundance of positrons (23%) and abundant high-energy γ photons that can lead to poor PET image quality. Its long half-life, low positron yield, high positron energy and abundant high energy γ emissions result in a high radiation dose and less-than-ideal imaging properties (as compared to other PET isotopes such as ^18^F).

Pharmacokinetics and radiation emission properties affect the choice of radionuclides for NIS reporter gene imaging. Use of radioiodine in the context of NIS reporter gene imaging with PET or SPECT is complicated by the fact that, as well as being a substrate of NIS, iodide is a substrate of the peroxidase machinery of thyroid hormone synthesis, leading to metabolic trapping. This trapping mechanism is an advantage in imaging normal thyroid function or response to thyroid ablation, but complicates reporter gene imaging because it increases thyroid uptake, making the tracer less available for uptake in the non-thyroidal target cells, which do not have this additional metabolic trapping machinery. In contrast, pertechnetate is a ‘pure’ NIS substrate without metabolic complications and therefore has reduced uptake in normal thyroid. A positron-emitting functional analog of ^99m^TcO_4_^−^ would bring the advantages of PET, including improved resolution, sensitivity, quantification and dynamic imaging, without the undesirable metabolic complications associated with [^124^I]iodide. We recently reported that [^18^F]BF_4_^−^ meets this need. It has the advantages of the functional analogy to ^99m^TcO_4_^−^^23–25^, while incorporating the radionuclide ^18^F, which is readily available and has excellent PET imaging characteristics.

## Results

### Specific [^18^F]BF_4_^−^ uptake in tumor cells expressing the NIS-TagRFP reporter

**Figure 1:**
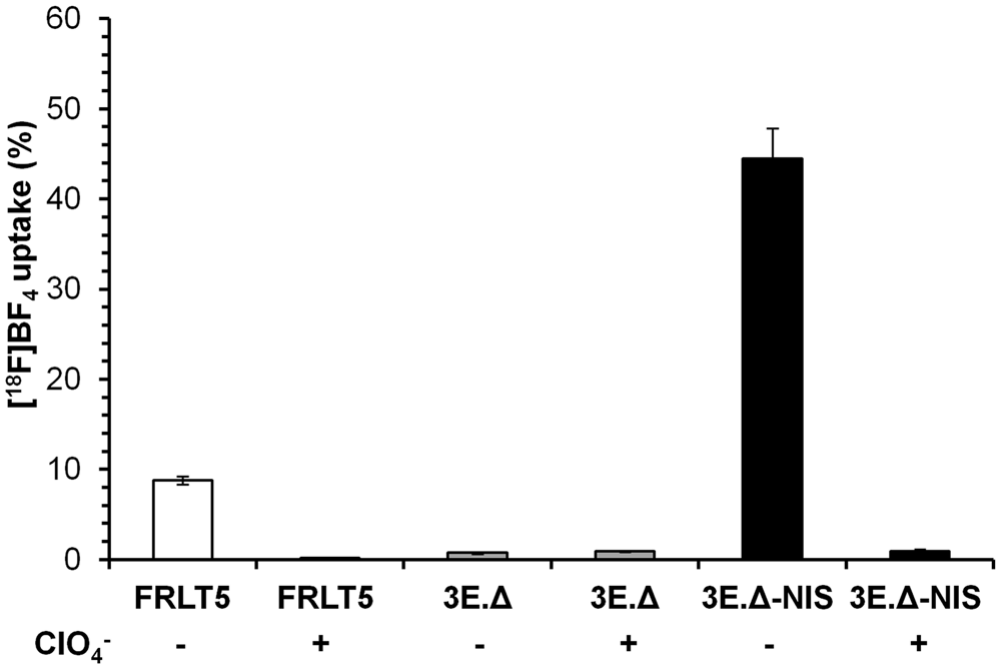
The exogenously expressed NIS reporter is functional in highly invasive breast carcinoma cells. [^18^F]BF_4_^−^ uptake was measured *in vitro* in highly metastatic cell lines either stably expressing the reporter gene NIS-TagRFP (3E.Δ-NIS, black bars) or not (3E.Δ). Uptake was compared to that of FRLT-5 thyroid cells, which endogenously express NIS if grown in the presence of TSH (see Materials and Methods). Perchlorate blockade was used to demonstrate uptake specificity. Error bars represent SD of three independent experiments, each in triplicate.

We chose the established breast adenocarcinoma cell line MTLn3E as the basis for the tumor models in this study. MTLn3E cells were engineered to stably and uniformly express (i) the truncated chemokine CXC receptor 4 to render the cells more metastatic^26, 27^ (3E.Δ cells), and (ii) the radionuclide-fluorescence fusion reporter gene NIS-TagRFP (3E.Δ-NIS cells)^28^. First, we determined the ability of these cell lines to take up the NIS PET radiotracer [^18^F]BF_4_^−^. As expected, 3E.Δ-NIS cells took up [^18^F]BF_4_^−^ significantly better than 3E.Δ cells, which lack the NIS-TagRFP reporter gene and any detectable endogenous NIS expression (59-fold difference; *P* = 0.0001; Fig. 1). 3E.Δ-NIS cells showed about 5-times higher [^18^F]BF_4_^−^ uptake than the TSH-treated rat thyroid cells (FRTL-5), which served as a reference. Pre-incubation of 3E.Δ-NIS cells with the competitive NIS substrate ClO_4_^−^ (1 μM) led to a 98% reduction (*P* = 0.0001) of [^18^F]BF_4_^−^ uptake thereby demonstrating that the uptake was NIS-specific (Fig. 1). We then determined the detection sensitivity of our nanoPET/CT *in vivo* imaging system by embedding NIS-positive 3E.Δ-NIS cells within small cell pellets of NIS-negative 3E.Δ cells. This revealed a detection limit of 2,000 3E.Δ-NIS cells within a volume of 500,000 cells (see Supplementary Fig. S1).

### Specific [^18^F]BF_4_^−^ uptake in tumors expressing the NIS-TagRFP reporter *in vivo*

**Figure 2:**
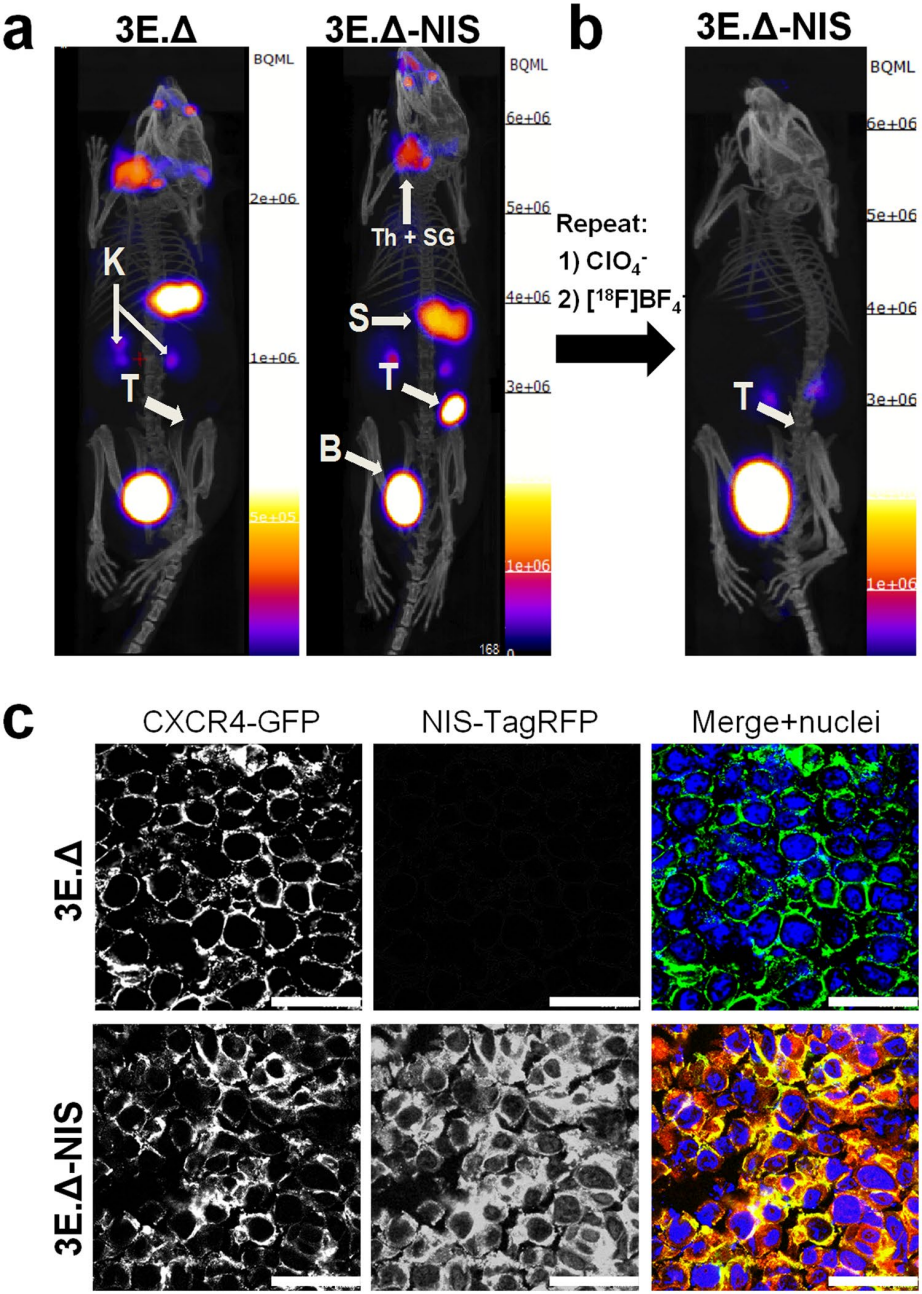
Primary xenograft tumors established from NIS-expressing breast cancer cells can specifically be imaged with [^18^F]BF_4_^−^-PET. (**a**) Tumors were established from 3E.Δ cells stably expressing constitutively active truncated CXCR4-GFP but not NIS-TagRFP (left) or from 3E.Δ-NIS cells stably expressing both truncated CXCR4-GFP and NIS-TagRFP (right). Tumors were imaged by [^18^F]BF_4_^−^-PET when sizes of ~55 mm^3^ were reached (see Supplementary Fig. S2A). As expected, no tumor signals were seen by PET/CT in the case of 3E.Δ tumors. For 3E.Δ-NIS tumors, PET/CT signals were very pronounced. A representative example is shown. (**b**) NIS specificity was tested in the same animals 48 h later by pre-treatment with perchlorate before repeat [^18^F]BF_4_^−^-PET imaging. All [^18^F]BF_4_^−^-PET signals except in the organs of the [^18^F]BF_4_^−^ excretion pathway, *i.e*. kidneys and bladder, vanished in perchlorate-treated animals (as compared to the earlier image of the same animal as in (**a**)), thereby proving successful NIS blockade and thus NIS specificity. All images are maximum intensity projections overlaid on CT. Abbreviations are: bladder (B), kidney (K), stomach (S), thyroid and salivary glands (Th + SG), and primary tumor (T). (**c**) Typical confocal micrographs of sections cut from primary tumors after γ-counting confirming expression of truncated CXCR4-GFP in both tumors but expression of NIS-TagRFP only in the 3E.Δ-NIS tumor (bottom row). Scale bars are 25 μm.

Subsequently, we established orthotopic tumor models using both 3E.Δ and 3E.Δ-NIS cell lines via mammary fat pad injections into young adult immunocompromised SCID/Beige mice. Tumors were grown to volumes of ~55 mm^3^ (Supplementary Fig. S2A), which was at a sufficiently early progression stage that metastasis on-set had not yet taken place in this model (as confirmed by animal dissection under fluorescence light). Animals bearing 3E.Δ-NIS tumors showed [^18^F]BF_4_^−^ uptake in the primary tumors and organs endogenously expressing NIS (thyroid and salivary glands, stomach, lachrymal glands; Fig. 2a/b) as determined by PET/CT imaging. [^18^F]BF_4_^−^ was also excreted renally as demonstrated by radiotracer amounts residing in the kidneys and the bladder with the majority in the latter at this imaging time point. NIS-specificity of [^18^F]BF_4_^−^ uptake was demonstrated *in vivo* by the following observations: first, there was no significant tracer uptake in non-NIS expressing primary tumors (3E.Δ) (Fig. 2a) and second, administration of ClO_4_^−^ before radiotracer administration in a repeat scan of 3E.Δ-NIS tumor-bearing animals abolished uptake in primary tumors and all endogenous NIS-expressing organs (Fig. 2b). Subsequent to animal euthanasia, primary tumors were harvested, sectioned and subjected to confocal fluorescence microscopy, which revealed the presence of green fluorescent tumor cells in both tumor groups (3E.Δ and 3E.Δ-NIS expressing Δ34-CXCR4-eGFP). Importantly, it also revealed expression and plasma membrane localization (required for function) of the reporter gene NIS-TagRFP in primary tumors grown from 3E.Δ-NIS cells only (Fig. 2c). The chosen sequential imaging approach in which one or more tracers are used in the same animal one after the other, after a suitable period for excretion and decay, relies on the assumption that the first tracer does not alter the biodistribution of the second. This assumption is experimentally validated (Fig. S3).

### Comparison of dynamic *in vivo* [^18^F]BF_4_^−^-PET with [^123^I]iodide-SPECT imaging

**Figure 3:**
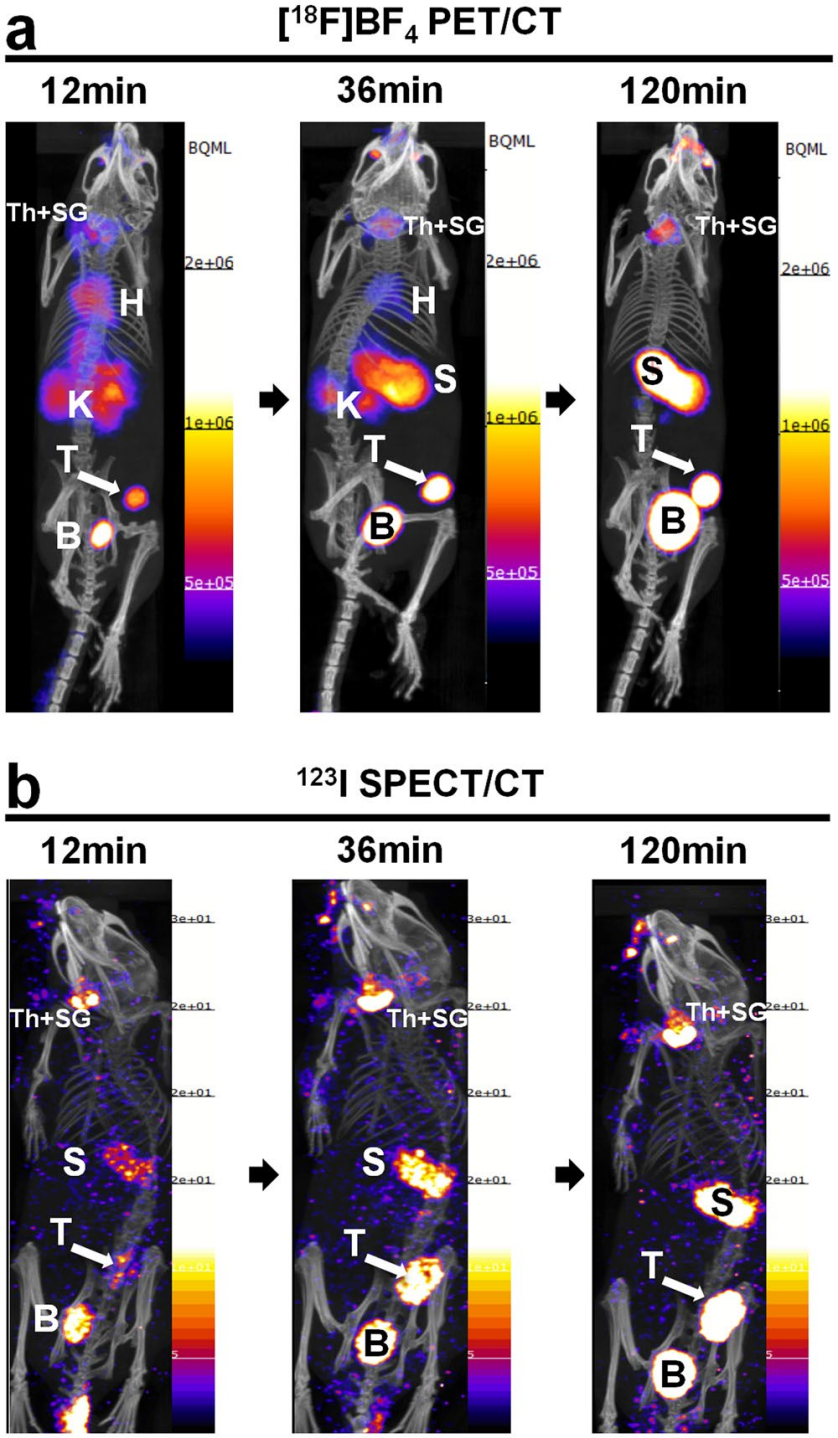
Comparative *in vivo* NIS imaging indicates differences between the PET tracer [^18^F]BF_4_^−^ and the SPECT tracer [^123^I]iodide. Tumors were established as in Fig. 2 but grown to sizes of ~350 mm^3^ (see Supplementary Fig. S2B) before being imaged by either [^18^F]BF_4_^−^-PET (**a**) or [^123^I]iodide-SPECT (**b**). Dynamic PET data were binned into time intervals comparable to sequential SPECT images (see Materials and Methods). Al images are maximum intensity projections overlaid on CT. Abbreviations are: bladder (B), heart (H), kidney (K), stomach (S), thyroid and salivary glands (Th + SG), and primary tumor (T). Representative images of cohorts of N = 3 are shown.

**Figure 4:**
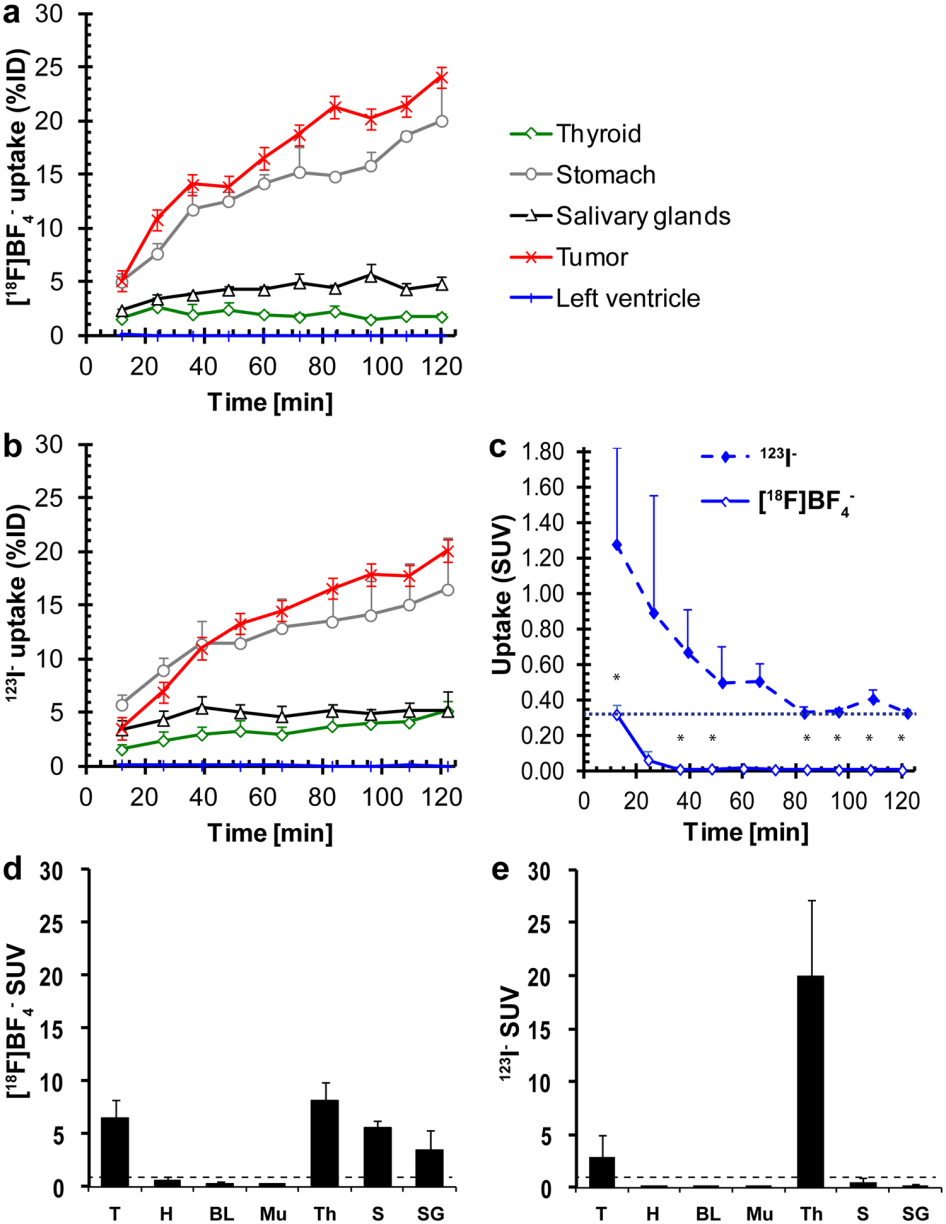
Quantitative analysis of *in vivo* NIS imaging and *ex vivo* biodistribution data reveals differences in pharmacokinetics between the PET tracer [^18^F]BF_4_^−^ and the SPECT tracer [^123^I]iodide. (**a/b**) Image data analysis of (**a**) dynamic [^18^F]BF_4_^−^-PET and (**b**) sequential [^123^I]iodide-SPECT experiments was performed as described in Materials and Methods. Cumulative data of organs expressing exogenous human NIS (primary tumor, red) and expressing endogenous NIS (thyroid, green; salivary glands, black; stomach, grey) are shown for each tracer. A heart volume comprising of the left ventricle was used as a surrogate for the blood pool (blue). Tracer batches were identical for all animals and experiments performed on the same day (N = 3 animals for each tracer). (**c**) A more detailed view of the radioactivity time course in the left ventricle (surrogate for the blood pool *in vivo*). It demonstrates the faster and more complete clearance of [^18^F]BF_4_^−^ as compared to [^123^I]iodide. (**d/e**) *Ex vivo* radioactivity analysis by γ-counting of harvested tissues was performed subsequent to imaging. Cumulative SUV data of all experimental animals of the [^18^F]BF_4_^−^ (**d**) and [^123^I]iodide (**e**) cohorts (N = 5 each). Abbreviations are (from left to right): primary tumors (T), heart (H), blood (BL), muscle (Mu), thyroid (Th), stomach (S), and salivary glands (SG). Error bars in all panels represent SD.

For this experiment primary 3E.Δ-NIS tumors were grown to sizes of ~350 mm^3^ (see Supplementary Fig. S2B) before imaging the animals by PET/CT (with [^18^F]BF_4_^−^ as the radiotracer) or by SPECT/CT (with [^123^I]iodide). PET images were acquired continuously (see Materials and Methods) while SPECT required a series of static images (12 min each). To render PET and SPECT scans comparable, we re-binned dynamic PET scans to the individual times required for short SPECT scans. Representative *in vivo* images are shown in Fig. 3a ([^18^F]BF_4_^−^-PET) and Fig. 3b ([^123^I]iodide-SPECT), respectively. The corresponding imaging experiments were also used to determine time-activity curves (TAC) for both tracers in the primary tumors and a variety of organs of interest including the thyroid, salivary glands, stomach and the left ventricle of the heart (as a surrogate for the blood pool). The TACs were determined by analyzing the respective VOIs from both [^18^F]BF_4_^−^-PET and [^123^I]iodide-SPECT scans (Fig. 4). [^18^F]BF_4_^−^ accumulated in the primary tumor and was readily detectable there (Fig. 3a) by 12 min after tracer administration (5.1 ± 0.3%ID; SUV = 3.1 ± 0.2; Fig. 4a). In contrast, due to the relatively high background/blood pool activity (see below), the [^123^I]iodide-SPECT images did not show a well-defined primary tumor 12 min after tracer administration (Fig. 3b). At that time point [^123^I]iodide uptake was 3.6 ± 0.7%ID (SUV = 2.2 ± 0.5) and also significantly lower than with [^18^F]BF_4_^−^ (*P* = 0.0290; Fig. 4b). To fully identify the whole primary tumor in [^123^I]iodide-SPECT images at least 36 min of post-administration imaging was required (Fig. 3b). We imaged each animal for a total period of 2 h after which the tumor uptake was 24.1 ± 3.3%ID (SUV = 14.8 ± 2.0) for [^18^F]BF_4_^−^ and 20.1 ± 2.8%ID (SUV = 12.4 ± 1.7) for [^123^I]iodide, respectively (Fig. 4a/b).

As expected, [^18^F]BF_4_^−^ accumulated also in organs endogenously expressing NIS. In the thyroid, its accumulation peaked after around 24 min at 2.6 ± 1.0%ID (SUV = 7.0 ± 2.5; Fig. 4a) before gradually decreasing again. In contrast, [^123^I]iodide continued to accumulate in the thyroid over the whole observation time. While its uptake levels were 1.6 ± 0.5%ID (SUV = 4.1 ± 1.2) at 12 min, they increased to 5.2 ± 0.9%ID (SUV = 13.3 ± 2.4) after 2 h (Fig. 4b). This demonstrated clearly different uptake behaviors of radioiodide and [^18^F]BF_4_^−^, consistent with the fact that [^123^I]iodide accumulates due to uptake and subsequent organification in the thyroid tissues, while [^18^F]BF_4_^−^ cannot be organified.

Importantly, clearance from the blood also differed between the two NIS radiotracers. The %ID values for the left ventricle of the heart were obtained directly from images and served as a surrogate for the blood pool. Interestingly, [^18^F]BF_4_^−^ cleared faster from circulation than [^123^I]iodide: 12 min after [^18^F]BF_4_^−^ administration we found it at levels of 0.10 ± 0.01%ID (SUV = 0.32 ± 0.05) while we found [^123^I]iodide at higher levels of 0.18 ± 0.07%ID (SUV = 1.28 ± 0.54; *P* = 0.0383; Fig. 4). At later time points both values decreased but in the case of [^123^I]iodide a plateau was reached (time > 80 min: 0.05 ± 0.01%ID; SUV = 0.33 ± 0.07), while for [^18^F]BF_4_^−^ the values approximated zero (%ID < 0.01; SUV ≤ 0.01; Fig. 4c). The differences between the two NIS radiotracers were significant (*P* < 0.05) for all time points ≥36 min.

An important consequence of the faster and more complete clearance of [^18^F]BF_4_^−^ paired with its faster tumor uptake is that its tumor-to-blood ratio (left ventricle as a surrogate) is higher as compared to [^123^I]iodide. We found it to be 51 and 3600 at 12 min and 36 min after [^18^F]BF_4_^−^ administration, which was 2.6- and 30.8-fold higher than for [^123^I]iodide (tumor-to-blood ratios of 20 and 117 at these times points, respectively). Because of [^123^I]iodide not being completely cleared from the blood stream or, potentially, organified ^123^I being secreted back into the blood stream, tumor-to-blood ratios were consistently lower at all later time points (18- to 25-fold).

Subsequent to *in vivo* imaging, animals were euthanized and organs harvested to determine the radioactivity taken up into the various tissues *ex vivo* by γ-counting. The average tumor SUV was higher in the case of [^18^F]BF_4_^−^ (6.4 ± 1.7; Fig. 4d) than in the case of [^123^I]iodide (2.9 ± 2.0; *P* = 0.0372). In contrast, the SUV values for the thyroid were lower for [^18^F]BF_4_^−^ (8.1 ± 2.1) than for [^123^I]iodide (20.0 ± 7.1; *P* = 0.0183) showing a similar trend as the data on thyroid uptake obtained by imaging (Fig. 4e).

### Small metastases of NIS-expressing tumors were detected by [^18^F]BF_4_^−^-PET but not by [^18^F]FDG-PET

**Figure 5:**
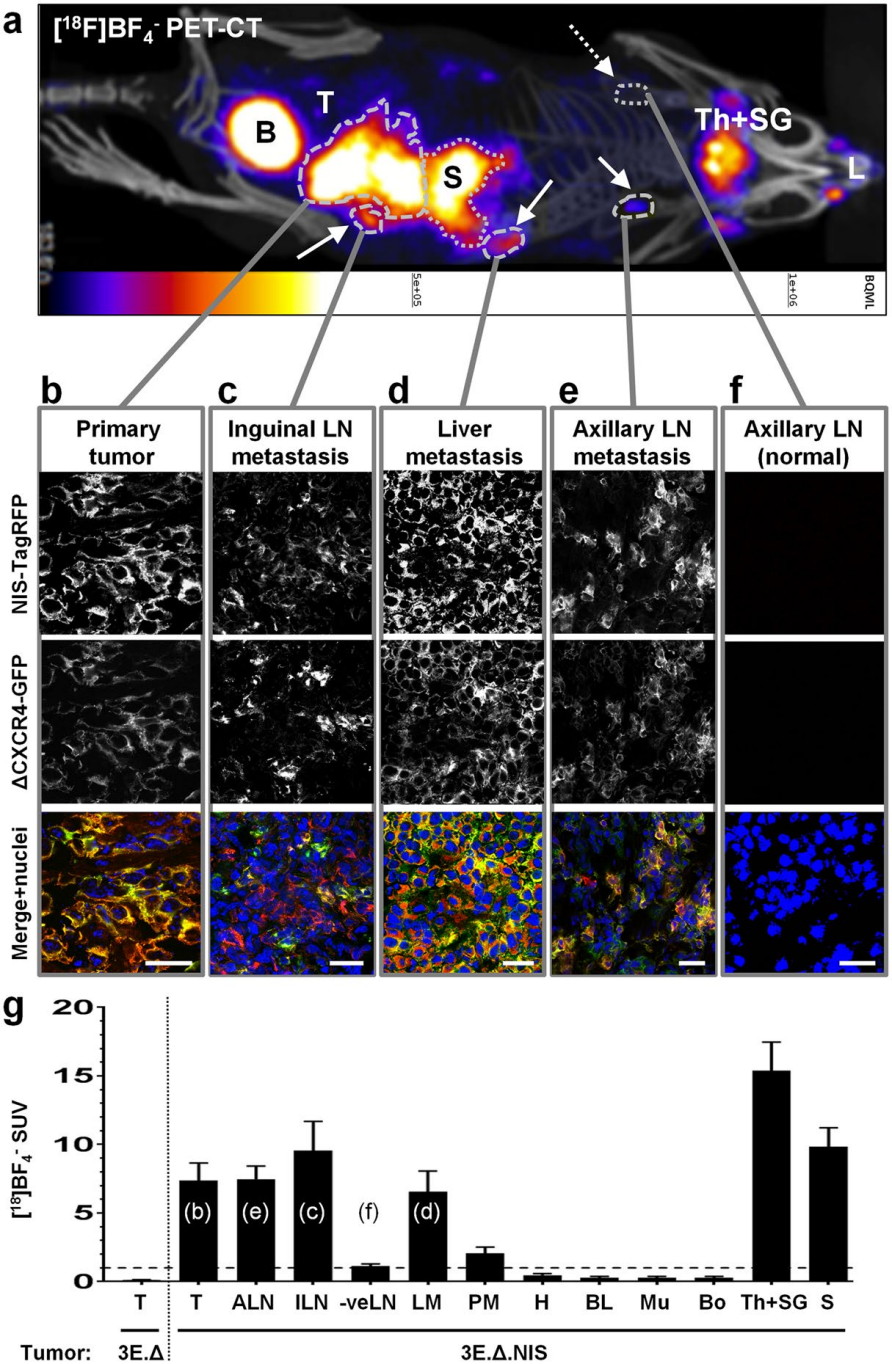
Combination of a NIS-expressing tumor model with [^18^F]BF_4_^−^-PET enables reliable and sensitive metastasis detection. (**a**) Representative maximum intensity projection image (overlaid on CT) of a [^18^F]BF_4_^−^-PET/CT scan of an animal growing a primary tumor established from 3E.Δ-NIS cells. Abbreviations are: bladder (B), lachrymal glands (L), stomach (S; dotted), thyroid and salivary glands (Th + SG), and primary tumor (T). Full arrows point to metastatic sites and include the following objects (dashed): left inguinal LN, liver metastasis, left axillary LN. Dotted arrow points to the site of the right axillary LN (no PET signal) and was later confirmed to be metastasis-free (see f). (**b–f**) Positivity/negativity for metastasis was determined from the PET/CT scans and tissues were subsequently harvested, γ-counted and sectioned; confocal microscopy confirmed the PET-based classifications in all cases. Confocal fluorescence microscopy revealed cancer cell presence by virtue of the fluorescence signals of NIS-TagRFP (top) and truncated CXCR4-GFP (middle). Data show the expected plasma membrane localization of both fusion proteins, thereby identifying cancer cells, and that signals belong to intact cells (membrane signals circumscribe Hoechst33342-stained cell nuclei (bottom)). Micrographs shown belong to the representative animal from (**a**) and represent (**b**) the primary tumor, (**c**) metastasis in the inguinal LN, (**d**) metastasis in the liver, (**e**) metastasis in the axillary LN, and (**f**) the contralateral axillary LN, which was not affected by metastasis (no signals in the [^18^F]BF_4_^−^-PET and fluorescence microscopy data). Scale bars are 25 μm. (**g**) [^18^F]BF_4_^−^ biodistribution data expressed as SUV as determined by γ-counting post tissue harvesting. Abbreviations are (from left to right): primary tumors (T), axillary lymph nodes (cancer cell-positive; ALN), inguinal lymph nodes (cancer cell-positive; ILN), metastasis-free lymph nodes (-veLN), liver metastases (LM), peritoneal metastases (PM), heart (H), blood (BL), muscle (Mu), bone (whole femur; Bo), thyroid and salivary glands combined (Th + SG), and stomach (S). Shown are pooled data of 3E.Δ-NIS tumor (N = 8, right of dotted line) and 3E.Δ tumor animals (NIS-negative tumors, N = 6). Error bars represent SEM. Lower case letters in brackets relate the figure panels (b–f) to the columns in which they were quantified together with equivalent tissues from other animals.

Next, we determined whether [^18^F]BF_4_^−^ would be a suitable PET radiotracer for metastasis tracking in NIS-expressing tumors. We established 3E.Δ-NIS tumors and let them progress until distant metastasis was a consistent feature (19 days; as determined by preliminary experiments based on animal dissection under fluorescence illumination). A representative [^18^F]BF_4_^−^-PET/CT image demonstrates radiotracer uptake in the primary tumor and various metastatic lesions (Fig. 5a). All regions of interest that were PET-positive were harvested. As lymph node metastasis was a predominant feature in this model, we also harvested lymph nodes with no PET-detectable uptake as controls, *i.e*. the lymph nodes contralateral to the affected ones from the same animals. Confocal fluorescence microscopy confirmed that all tissues with detectable radiotracer uptake in the [^18^F]BF_4_^−^-PET/CT images were also positive for 3E.Δ-NIS cells (N = 8 animals; see Fig. 5b–e for a representative example). Conversely, no 3E.Δ-NIS cells were detected in contralateral lymph nodes that did not show any uptake in the corresponding [^18^F]BF_4_^−^-PET images (Fig. 5f). The average SUV values in the primary tumors and all metastases were greater than one (Fig. 5g). For example, metastasis-containing axillary and inguinal lymph nodes had average SUV values of 7.5 ± 2.6 (N = 7) and 9.6 ± 6.0 (N = 8), which were significantly higher than for unaffected lymph nodes (1.1 ± 0.63; *P* < 0.0001; N = 17). Importantly, all [^18^F]BF_4_^−^-PET-negative lymph nodes had average SUV values of ~1 as was the case for other non-cancerous tissues lacking endogenous NIS expression. Peritoneal metastases were not as frequent (20–30% of mice) as compared to inguinal and axillary lymph node metastases (100% and 80–90% of mice, respectively) and were also smaller. Peritoneal metastases showed the lowest SUV values of all cancerous tissues (2.1 ± 0.8; N = 3) but were still significantly different from metastasis-free tissues (*P* = 0.0386 as compared to lymph nodes and *P* = 0.0003 as compared to muscle); we suspect this was due to these metastases being inseparable by dissection from mesenteric tissues thus, likely resulting in an overestimation of their weights and a consequential underestimation of their SUV values. It is also noteworthy, that tumors established from 3E.Δ cells had very low SUV values (0.11 ± 0.06; N = 6), which were significantly different from those of 3E.Δ-NIS tumors (N = 8; *P* = 0.0004) (Fig. 5g). This not only demonstrated the performance of the reporter gene *per se*, but also showed that the enhanced permeability and retention effect plays no role in this experimental setting.

**Figure 6:**
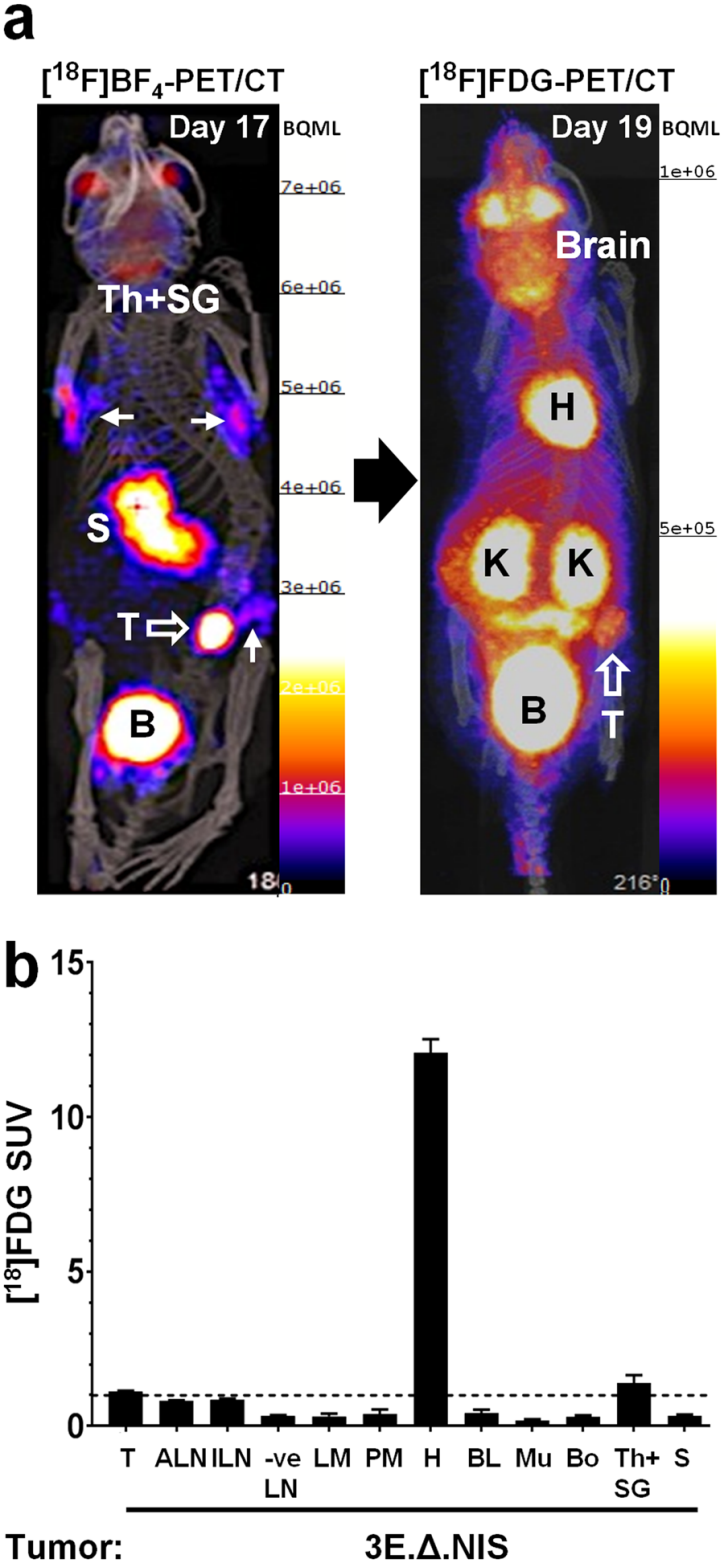
Metastasis detection with [^18^F]BF_4_^−^-PET is superior to metabolic imaging by [^18^F]FDG-PET in the NIS-expressing tumor model. 3E.Δ-NIS tumors were established and grown to sizes of ~350 mm^3^ before being imaged (see Materials and Methods). (**a**, left) Animals were first imaged by [^18^F]BF_4_^−^-PET. A maximum intensity projection (overlaid on CT) is shown. Abbreviations are: bladder (B), stomach (S), thyroid and salivary glands (Th + SG), and primary tumor (T); open arrow). Full arrows point to metastatic sites, which include the following objects in this animal (from left to right and top to bottom): right axillary LN, left axillary LN, left inguinal LN (draining LN of the primary tumor). (**a**, right) The animals were allowed to recover after imaging and the radioactivity was allowed to decay (48 h) before metabolic imaging with [^18^F]FDG-PET was performed on the same animals. A maximum intensity projection (overlaid on CT) of the same animal as in (**a**) is shown. Abbreviations are: bladder (B), heart (H), kidneys (K), and primary tumor (T; open arrow). Signal-to-background is insufficient to identify any of the metastatic sites previously identified in (**a**, left). (**b**) [^18^F]FDG biodistribution data expressed as SUV as determined by γ-counting post tissue harvesting. Abbreviations are (from left to right): primary tumors (T), axillary lymph nodes (cancer positive; ALN), inguinal lymph nodes (cancer cell positive; ILN), metastasis-free lymph nodes (-veLN), liver metastases (LM), peritoneal metastases (PM), heart (H), blood (BL), muscle (Mu), bone (whole femur; Bo), thyroid and salivary glands combined (Th + SG), and stomach (S). Shown are pooled data (N = 8 animals) and error bars represent SEM. While lymph node metastasis could not be identified by imaging (**a**, right), *ex vivo* γ-counting allowed the differentiation of LNs that contained cancer cells (ALN, ILN) from metastasis-free LNs (-veLN), albeit with a small dynamic range. Liver (LM) and peritoneal (PM) metastases were not distinguishable from metastasis-free organs by *ex vivo* γ-counting.

To compare NIS-afforded metastasis tracking by [^18^F]BF_4_^−^-PET with the performance of [^18^F]FDG for the same purpose^29^, mice with 3E.Δ-NIS tumors were first imaged by [^18^F]BF_4_^−^-PET/CT (day 17) and, after complete ^18^F decay (1.3·10^−6^% residual ^18^F radioactivity after 48 h), with [^18^F]FDG-PET/CT (day 19). [^18^F]BF_4_^−^-PET images (Fig. 6a, left) showed radiotracer uptake in the primary tumors and axillary and inguinal lymph nodes, all of which were later confirmed *ex vivo* to contain metastases using confocal fluorescence microscopy. In contrast, [^18^F]FDG-PET imaging (Fig. 6a, left) allowed the image-based detection of primary tumors but not metastases (Fig. 6a, right). Subsequent to [^18^F]FDG-PET imaging, the animals were euthanized and tissues harvested for *ex vivo* γ-counting. Average SUV values of primary tumors were relatively low 1.1 ± 0.10 (N = 8 animals) but significantly higher than the SUV values for blood (0.41 ± 0.35; *P* < 0.0001) or muscle (0.18 ± 0.12; *P* < 0.0001). Average SUV values for metastasis-positive axillary (0.82 ± 0.04) or inguinal (0.86 ± 0.10) lymph nodes were small but higher than SUV values of metastasis-free lymph nodes (0.33 ± 0.14; *P* < 0.0001). However, smaller metastases such as those in the liver (SUV 0.31 ± 0.16) and the peritoneum (SUV 0.39 ± 0.21) were indistinguishable from metastasis-free lymph nodes (*P* = 0.8208 and *P* = 0.5708, respectively) also by *ex vivo* γ-counting. Generally, this was in marked contrast to the [^18^F]BF_4_^−^-PET data (Fig. 5g) where metastasis-positive lymph nodes differed significantly in their SUV values (6.5- to 8.5-fold increase) from metastasis-free lymph nodes, indicating not only better sensitivity but also better dynamic range for [^18^F]BF_4_^−^-PET as compared to [^18^F]FDG-PET in this model.

## Discussion

Cell tracking is a task that involves differentiating the cells of interest (e.g. cancer cells) from all other cells of the body in space and over an extended period of time, which requires excellent contrast. Some intrinsic properties of cancer cells, for example their elevated metabolic activity can be exploited to generate contrast (e.g. by using FDG). In principle, the sensitivity of pre-clinical radionuclide imaging-afforded cell tracking is dependent on multiple parameters. These include high expression levels of the tracer target in the cells of interest, low target abundance in other host tissues, adequate tracer affinity, uptake and retention, radionuclide properties, and the chosen imaging technology. We have previously demonstrated the utility of a NIS-based radionuclide-fluorescence imaging strategy for *in vivo* metastasis tracking and therapeutic studies in a breast cancer xenograft mode^28^. In this previous work we used ^99m^TcO_4_^−^ as a radiotracer for SPECT imaging as radiotracers for PET imaging were either a scarce resource at the time or showed sub-optimal properties (*e.g*. [^124^I]iodide^22^). Here, we evaluated our recently introduced PET tracer [^18^F]BF_4_^−^ for *in vivo* metastasis imaging and compared its metastasis detection capability to metabolic PET imaging afforded by [^18^F]FDG in the same NIS-expressing tumor model.

For sensitive imaging, the pharmacokinetics of a radiotracer can have a decisive impact on the imaging/detection performance. Hence, we were interested in a pharmacokinetic comparison of tetrafluoroborate- and iodide-based radiotracers. Our comparison of [^18^F]BF_4_^−^ with [^123^I]iodide showed that several pharmacokinetic features render [^18^F]BF_4_^−^ superior to radioiodide for sensitive imaging in NIS-reporter gene expressing cell tracking applications:

Firstly, the absolute thyroid uptake is greater for [^123^I]iodide; thyroid is the major sink for circulating [^123^I]iodide, which is metabolically trapped by conversion into thyroid hormones once transported into the thyroid^7^. In contrast, [^18^F]BF_4_^−^ is not metabolically trapped in the thyroid. Consequently, [^18^F]BF_4_^−^ is more available than radioiodide for uptake into other NIS expressing tissues (this may also account for the higher stomach uptake of [^18^F]BF_4_^−^; see Fig. 4d/e).

Secondly, NIS reporter gene-expressing cancerous tissues lack the metabolic entrapment features of the thyroid. As a consequence, the advantage that radioiodide has over [^18^F]BF_4_^−^ for thyroid imaging is irrelevant for NIS reporter gene imaging^30^. We also found that tetrafluoroborate is taken up by human non-thyroidal NIS-expressing cells with higher affinity (as reflected in lower IC_50_) than iodide as demonstrated in a [^99m^Tc]pertechnetate competition experiment (5.6-times smaller IC_50_; see Supplementary Fig. S4). Both higher availability and better cellular uptake contribute to a higher absolute uptake of [^18^F]BF_4_^−^ as compared to [^123^I]iodide (see Fig. 4d/e) in non-thyroidal NIS-expressing tissues. It is noteworthy that we cannot completely rule out that there is a difference in relative affinity of iodide and BF_4_^−^ between human NIS (ectopically expressed by the reporter gene-expressing tumor cells) and mouse NIS (expressed endogenously), which could contribute, at least in part, to our findings. More cell biological experiments dedicated to radiotracer affinity and uptake kinetics in cells expressing mouse or human NIS variants are required to elucidate this and are subject of future work.

Thirdly, we observed that [^18^F]BF_4_^−^ is cleared faster and more completely from the blood than [^123^I]iodide (Fig. 4c). [^123^I]iodide is incorporated into hormones within the thyroid ^7^ and it is not unlikely that a certain portion of these hormones is secreted back into circulation^31, 32^, thereby contributing to lower apparent clearance of ^123^I from the blood stream. It may be that [^18^F]BF_4_^−^ is more efficiently extracted and excreted by the kidneys than [^123^I]iodide, but more studies will be required to distinguish the precise mechanistic reasons for these apparent differences in clearance rates. Regardless of the mechanism, the faster and more complete blood clearance contributes to higher target-to-background ratios in non-thyroidal NIS expressing tissues for [^18^F]BF_4_^−^ than for [^123^I]iodide (18- to 30-fold better tumor-to-blood pool surrogate in the case of [^18^F]BF_4_^−^ depending on the imaging time after tracer administration).

[^18^F]BF_4_^−^ uptake in NIS expressing organs and lesions stemming from our NIS reporter gene-expressing breast cancer cells was specific and confirmed *ex vivo* by radioactivity measurements in individual tissues (Figs 2 and 5). *In vivo*, [^18^F]BF_4_^−^ enabled detection of primary tumors but, importantly, also small metastatic lesions. [^18^F]BF_4_^−^-PET-positive lesions were analyzed by immunofluorescence microscopy for cancer cell presence, which was confirmed in all cases (N = 20 different metastatic tissues from eight different animals; Fig. 5). Lymph nodes were the expected sites of metastasis in this model; all lymph nodes found to be positive by imaging contained cancer cells and *vice versa*, all lymph nodes in which we found cancer cells were also detected by imaging that preceded tissue harvesting and histology. We also analyzed [^18^F]BF_4_^−^-PET-negative lymph nodes using *ex vivo* immunofluorescence microscopy and did not find any cancer cells in those tissues (N = 17 different metastasis-free axillary and inguinal lymph nodes from eight animals; Fig. 5). These findings were in agreement with our previous study establishing NIS reporter gene imaging as an excellent strategy for *in vivo* metastasis tracking by SPECT imaging using ^99m^TcO_4_^−^. The sensitivity of our [^18^F]BF_4_^−^-PET imaging approach *in vitro* was ~2000 NIS-expressing cells in a volume of 0.5 million NIS-negative cells (Supplementary Fig. S1) and also comparable to our previous [^99m^Tc]pertechnetate-SPECT experiments^28^.

We were further interested in comparing this [^18^F]BF_4_^−^-PET imaging approach to routine metabolic imaging by [^18^F]FDG-PET in our NIS expressing tumor model. We found the SUV values for [^18^F]BF_4_^−^ to be significantly larger as compared to those obtained by use of [^18^F]FDG in the same organs (compare Figs 5g with 6b). Importantly, metastasis detection by PET imaging could not be achieved with [^18^F]FDG-PET due to the shortcomings of [^18^F]FDG with respect to its contrast and specificity; other organs with high metabolic activities such as the heart and kidneys led to a deterioration of contrast in adjacent organs of interest such as the lung and lymph nodes. [^18^F]FDG heart uptake can, however, be suppressed (but not abolished) by fasting the animals prior to the administration of [^18^F]FDG^33^. While metabolic imaging by [^18^F]FDG-PET is more versatile in preclinical research in that it does not require tumor models genetically engineered to express a reporter gene such as NIS, it is of very limited use for metastasis imaging and longitudinal metastasis tracking and related drug and treatment development studies. In contrast, we demonstrated sensitive and specific metastasis detection by [^18^F]BF_4_^−^-PET imaging in a NIS-expressing tumor model.

*In vivo* cell tracking is currently a growing field, enabled by technological advances that improved detection limits, resolution and accessible information through multiplexing (enabled by multi-modality imaging). In this study, we apply this concept to track spontaneous cancer cell metastasis, also with a view to using this as an assay for the evaluation and validation of future anti-metastatic drug candidates. Importantly, our reporter gene:radiotracer pair for PET imaging (NIS:F-18-tetrafluoroborate) can also be used for different cell tracking applications. For example, cell therapies are currently emerging as promising therapeutic approaches not only for the treatment of cancer^34^ but also in the contexts of transplantation immunology^35^ and regenerative medicine^36, 37^. Whole-body *in vivo* imaging-afforded cell tracking applications are becoming increasingly important for the successful development and translation of such cell therapies, in particular in the contexts of therapy safety and monitoring.

## Conclusion

We successfully demonstrated the utility of the recently developed NIS radiotracer [^18^F]BF_4_^−^ for tumor and metastasis imaging by PET in a preclinical model of breast cancer. We demonstrated very high sensitivity and specificity of the approach and also provided an explanation as to why the pharmacokinetic properties of [^18^F]BF_4_^−^ are better suited for studying NIS-expressing tumor models than those of radioiodine. In addition, we demonstrated that the detectability of NIS-expressing cancer cells and consequently metastasis tracking is greatly enhanced when [^18^F]BF_4_^−^-PET is used as NIS tracer as compared to routine metabolic imaging afforded by [^18^F]FDG-PET. This will be particularly useful for challenging applications in cancer research (*e.g*. response-to-treatment studies, development of anti-metastatic therapies *etc*.). In addition, we believe that other fields in need of highly sensitive *in vivo* cell tracking tools are likely to benefit from this methodology (e.g. immunology and stem cell research).

## Materials and Methods

### Reagents and Cells

All chemicals and reagents were purchased from Fischer-Scientific (Loughborough, UK), Millipore (Watford, UK), Sigma (Gillingham, UK) or VWR (Lutterworth, UK) unless otherwise indicated. Fisher rat thyroid cells FRTL-5 were purchased from ATCC (*via* LGC Standards, Teddington, UK). Rat breast adenocarcinoma cells (MTLn3E) were a gift from Dr. Erik Sahai (London Research Institute) and were engineered to express the truncated version of the chemokine CXC receptor 4 (Δ34-CXCR4) fused to monomeric eGFP (cells named 3E.Δ) to obtain a highly metastatic cancer cell line^26^ that spontaneously metastasizes. These cells were further modified to stably express human NIS fused to monomeric TagRFP (cells named 3E.Δ-NIS) and NIS expression and function was confirmed previously^28^. For cell culture see Supplementary Information. [^123^I]iodide was purchased from GE Healthcare (Little Chalfont, UK). [^18^F]BF_4_^−^ was synthesized as previously described with minor modifications^23^ (see Supplementary Information).

### Cellular uptake of NIS tracers

Uptake experiments were performed as previously described^28^, but with [^18^F]BF_4_^−^ as a radiotracer (50 kBq per mL per 10^6^ cells). Uptake in the cells was compared to TSH-stimulated the FRTL-5 cells. For the comparison of relative tetrafluoroborate and iodide uptake into cells, we performed competition assays against [^99m^Tc]pertechnetate. Briefly, standard uptake assays (as previously described^28^) were performed in the presence of differing amounts of either cold tetrafluoroborate, cold iodide or [^99^Tc]pertechnetate (for assay details see Supplementary Fig. 3). The co-substrate effects on cellular [^99m^Tc]pertechnetate uptake were quantified and fitted to a sigmoid model using Prism software v7.01 (GraphPad, La Jolla, US).

### Animal tumor model

Young adult (5–6 weeks old) female SCID/Beige (CB17.Cg-*Prkdc* ^*scid*^ *Lyst* ^*bg*−*J*^/Crl; Charles River, UK) were a kind gift of Dr. Maher (King’s College London). All animals were housed within filter-top cages in Scantainer units in approved facilities and given food and water *ad libitum*. 3E.Δ.NIS cells were trypsinized, washed with pre-warmed Hank’s buffered saline (HBSS; without Ca^2+^ and Mg^2+^), re-suspended, counted, and aliquots of 5 × 10^5^ cells in 50 µL HBSS were injected subcutaneously into the left mammary fat pad between nipples four and five. Once palpable, tumor volumes were measured with calipers using the following formula; V = π/6·l·w·d, where l is length, w is width and d is depth. All animal experiments were approved by the UK Home Office and an ethical review panel and all requirements dictated by E.U. and UK legislation and the above committees were met.

### *In vivo* radionuclide imaging

Mice were anesthetized using isoflurane (2% (v/v) in O_2_) and radiotracers were injected into their tail veins; 5 MBq of [^18^F]BF_4_^−^ or [^18^F]FDG, or 15 MBq of [^123^I]iodide, each in 100 μL HBSS. In static scans, animals were sedated and imaged 40 min after radiotracer administration using nanoScanPET/CT (Mediso, Budapest, Hungary) or nanoSPECT/CT Plus (Mediso, Budapest, Hungary) instruments. Static PET and SPECT scans lasted 30 min each with CT image acquisition performed subsequent to radionuclide imaging. In dynamic scans, PET/CT acquisition was started before radiotracer administration and animals were imaged from the moment of radiotracer administration for 2 h. To make dynamic PET/CT data comparable to static SPECT/CT data, dynamic PET/CT data were binned into 12 min time intervals, and thereby rendered static to match the static time-series of the SPECT/CT data. PET imagereconstruction was performed using Tera-Tomo^TM^ 3D PET image reconstruction software (Mediso, Budapest, Hungary). SPECT/CT scans were performed sequentially for 12 min each and individually reconstructed over a period of 2 h. Decay correction for the different radioisotope half lives was done using the built-in features in the analysis software packages.

For specificity tests of the NIS radiotracer [^18^F]BF_4_^−^, animals were first imaged and then rested awake until the radioactivity had decayed sufficiently to be regarded as negligible, *i.e*. 48 h (1.3 · 10^−6^% residual ^18^F radioactivity). Subsequently, the competitive substrate perchlorate was administered at a dose of 200 mg/kg and 30 min later animals were re-imaged under the same conditions as described above. Importantly, repeat imaging with the same radiotracer was no affected by tracer amounts from the first imaging session (Fig. S3).

### Analysis of radioactivity from images and in harvested tissues

To quantify the radioactivity from images, 3D volumes of interest (VOI) enclosing the tumor, other organs of interest, or the whole mouse excluding the tail were drawn. The total activity in the whole animal (excluding the tail) at t = 0 was defined as the injected dose. Radioactivity in each VOI at each time point was measured and expressed as percent of injected dose (%ID). Each radiotracer time-activity curve (TAC) was an average of %ID of three xenograft-bearing animals. CT images were used to draw a VOI and provide the volumes required for SUV calculations from imaging data. A volume comprising of the left ventricle of the heart served as a surrogate for the blood pool with the procedure for obtaining corresponding TACs being described elsewhere^38^.

In metastases detection experiments the same animals were imaged via PET/CT with [^18^F]BF_4_^−^ and with [^18^F]FDG on days 17 and 19, respectively. Day 17 [^18^F]BF_4_^−^-PET/CT images were used to identify focal radiotracer uptake. Subsequently, tissues were harvested for *ex vivo* γ-counting ([^18^F]FDG biodistribution) using a 1282 Compugamma counter (LKB-Wallac, Australia). For the *ex vivo* comparison of [^18^F]BF_4_^−^ and [^18^F]FDG biodistributions, additional mice from the same experimental cohort (also imaged by [^18^F]BF_4_^−^-PET on day 17) were injected with [^18^F]BF_4_^−^ (but not [^18^F]FDG) on day 19, and tissues harvested and subjected to *ex vivo* γ-counting ([^18^F]BF_4_^−^ biodistribution).

For terminal *ex vivo* γ-counting, animals were euthanized one hour following radiotracer administration. All harvested tissues were γ-counted and their standardized uptake values (SUVs) calculated as previously described^28^. A minimum of four animals was used for this type of *ex vivo* analysis per experimental cohort. Furthermore, all harvested tissues were subjected to histologic analysis by confocal fluorescence microscopy.

### Histologic analysis by confocal fluorescence microscopy

Harvested tissues were embedded in optimal cutting temperature (OCT) medium and frozen in isopentane pre-cooled over liquid nitrogen and stored at −80 °C. 10 µm sections were cut with a Cryomatic cryostat (Bright Ltd, Huntingdon, UK), placed on Superfrost Plus slides and fixed with paraformaldehyde (1 mg/mL in HBSS) for 15 min at room temperature. Fixed slides were stained with Hoechst 33342 (1 µg/mL; Invitrogen, Paisley, UK) for 15 min at room temperature, washed with HBSS (3x for 5 min) and rinsed with deionized water before being mounted with Vectashield (Vectorlabs, Peterborough, UK). Images were taken with a TCS SP5II confocal fluorescence microscope (Leica, Wetzlar, Germany) equipped with a Plan-Apochromat 63x/1.4NA objective and filter sets appropriate for imaging Hoechst 33342 (cell nuclei), GFP (Δ34-CXCR4-GFP) and TagRFP (NIS-TagRFP). Images were acquired with the LAS-AF software, exported in TIFF file format and post-processed using ImageJ software v1.41 (NIH).

### Statistical analysis

Two-tailed unpaired Student’s *t*-tests (with alpha of 0.05) were used for comparisons between the two radiotracers. Sample sizes in comparative experiments were equal (unless otherwise indicated). Numbers in the text indicate means of pooled data ± standard deviation (SD) unless otherwise stated. All statistical data were calculated using Prism software v7.01 (GraphPad, La Jolla, CA/USA).

## Electronic supplementary material

Supplementary Information

## References

[1] Chaffer CL, Weinberg RA (2011). A perspective on cancer cell metastasis. Science.

[2] Lajtos I (2014). Cold wall effect eliminating method to determine the contrast recovery coefficient for small animal PET scanners using the NEMA NU-4 image quality phantom. Physics in medicine and biology.

[3] Nagy K (2013). Performance evaluation of the small-animal nanoScan PET/MRI system. J Nucl Med.

[4] Deleye S, Van Holen R, Verhaeghe J, Vandenberghe S, Stroobants S, Staelens S (2013). Performance evaluation of small-animal multipinhole muSPECT scanners for mouse imaging. European journal of nuclear medicine and molecular imaging.

[5] Brader P, Serganova I, Blasberg RG (2013). Noninvasive molecular imaging using reporter genes. J Nucl Med.

[6] Chung JK (2002). Sodium iodide symporter: its role in nuclear medicine. J Nucl Med.

[7] Portulano C, Paroder-Belenitsky M, Carrasco N (2014). The Na+/I- symporter (NIS): mechanism and medical impact. Endocr Rev.

[8] Groot-Wassink T, Aboagye EO, Wang Y, Lemoine NR, Keith WN, Vassaux G (2004). Noninvasive imaging of the transcriptional activities of human telomerase promoter fragments in mice. Cancer research.

[9] Chen L (2006). Radioiodine therapy of hepatoma using targeted transfer of the human sodium/iodide symporter gene. J Nucl Med.

[10] Sieger S (2003). Tumour-specific activation of the sodium/iodide symporter gene under control of the glucose transporter gene 1 promoter (GTI-1.3). European journal of nuclear medicine and molecular imaging.

[11] Chisholm EJ (2009). Cancer-specific transgene expression mediated by systemic injection of nanoparticles. Cancer research.

[12] Klutz K (2009). Targeted radioiodine therapy of neuroblastoma tumors following systemic nonviral delivery of the sodium iodide symporter gene. Clinical cancer research: an official journal of the American Association for Cancer Research.

[13] Merron A (2010). Assessment of the Na/I symporter as a reporter gene to visualize oncolytic adenovirus propagation in peritoneal tumours. European journal of nuclear medicine and molecular imaging.

[14] Merron A (2007). SPECT/CT imaging of oncolytic adenovirus propagation in tumours *in vivo* using the Na/I symporter as a reporter gene. Gene therapy.

[15] Dingli D, Kemp BJ, O’Connor MK, Morris JC, Russell SJ, Lowe VJ (2006). Combined I-124 positron emission tomography/computed tomography imaging of NIS gene expression in animal models of stably transfected and intravenously transfected tumor. Molecular imaging and biology: MIB: the official publication of the Academy of Molecular Imaging.

[16] Carlson SK (2009). Quantitative molecular imaging of viral therapy for pancreatic cancer using an engineered measles virus expressing the sodium-iodide symporter reporter gene. AJR American journal of roentgenology.

[17] Higuchi T (2009). Reporter gene PET for monitoring survival of transplanted endothelial progenitor cells in the rat heart after pretreatment with VEGF and atorvastatin. J Nucl Med.

[18] Terrovitis J (2008). Ectopic expression of the sodium-iodide symporter enables imaging of transplanted cardiac stem cells *in vivo* by single-photon emission computed tomography or positron emission tomography. Journal of the American College of Cardiology.

[19] Jung KH, Paik JY, Lee YL, Lee YJ, Lee J, Lee KH (2009). Trypsinization severely perturbs radioiodide transport via membrane Na/I symporter proteolysis: implications for reporter gene imaging. Nuclear medicine and biology.

[20] Ricci D (2008). Non-invasive radioiodine imaging for accurate quantitation of NIS reporter gene expression in transplanted hearts. European journal of cardio-thoracic surgery: official journal of the European Association for Cardio-thoracic Surgery.

[21] Cascini GL (2014). 124 Iodine: a longer-life positron emitter isotope-new opportunities in molecular imaging. Biomed Res Int.

[22] Pentlow KS (1996). Quantitative imaging of iodine-124 with PET. J Nucl Med.

[23] Jauregui-Osoro M (2010). Synthesis and biological evaluation of [(18)F]tetrafluoroborate: a PET imaging agent for thyroid disease and reporter gene imaging of the sodium/iodide symporter. European journal of nuclear medicine and molecular imaging.

[24] Weeks AJ, Jauregui-Osoro M, Cleij M, Blower JE, Ballinger JR, Blower PJ (2011). Evaluation of [18F]-tetrafluoroborate as a potential PET imaging agent for the human sodium/iodide symporter in a new colon carcinoma cell line, HCT116, expressing hNIS. Nuclear medicine communications.

[25] Khoshnevisan A (2016). [(18)F]tetrafluoroborate as a PET tracer for the sodium/iodide symporter: the importance of specific activity. EJNMMI Res.

[26] Vermeer LS, Fruhwirth GO, Pandya P, Ng T, Mason AJ (2012). NMR metabolomics of MTLn3E breast cancer cells identifies a role for CXCR4 in lipid and choline regulation. J Proteome Res.

[27] Ueda Y, Neel NF, Schutyser E, Raman D, Richmond A (2006). Deletion of the COOH-terminal domain of CXC chemokine receptor 4 leads to the down-regulation of cell-to-cell contact, enhanced motility and proliferation in breast carcinoma cells. Cancer research.

[28] Fruhwirth GO, Diocou S, Blower PJ, Ng T, Mullen GE (2014). A whole-body dual-modality radionuclide optical strategy for preclinical imaging of metastasis and heterogeneous treatment response in different microenvironments. J Nucl Med.

[29] Dose-Schwarz J (2010). Assessment of residual tumour by FDG-PET: conventional imaging and clinical examination following primary chemotherapy of large and locally advanced breast cancer. British journal of cancer.

[30] Daniels GH, Haber DA (2000). Will radioiodine be useful in treatment of breast cancer?. Nat Med.

[31] Rousset B, Mornex R (1991). The thyroid hormone secretory pathway–current dogmas and alternative hypotheses. Mol Cell Endocrinol.

[32] van der Deure WM, Peeters RP, Visser TJ (2010). Molecular aspects of thyroid hormone transporters, including MCT8, MCT10, and OATPs, and the effects of genetic variation in these transporters. J Mol Endocrinol.

[33] Fueger BJ (2006). Impact of animal handling on the results of 18F-FDG PET studies in mice. J Nucl Med.

[34] Couzin-Frankel J (2013). Breakthrough of the year 2013. Cancer immunotherapy. Science.

[35] Boardman, D. A. *et al*. Expression of a Chimeric Antigen Receptor Specific for Donor HLA Class I Enhances the Potency of Human Regulatory T Cells in Preventing Human Skin Transplant Rejection. *American Journal of Transplantation* **17**, 931–943 doi: 10.1111/ajt.14185 (2017). 10.1111/ajt.14185 28027623

[36] Rashid T, Takebe T, Nakauchi H (2015). Novel strategies for liver therapy using stem cells. Gut.

[37] Ellison GM (2013). Adult c-kit(pos) cardiac stem cells are necessary and sufficient for functional cardiac regeneration and repair. Cell.

[38] Locke LW, Berr SS, Kundu BK (2011). Image-derived input function from cardiac gated maximum a posteriori reconstructed PET images in mice. Molecular imaging and biology: MIB: the official publication of the Academy of Molecular Imaging.

