# Supplementary material for: [18F]tetrafluoroborate-PET/CT enables sensitive tumor and metastasis in vivo imaging in a sodium iodide symporter-expressing tumor model
Source: Sci Rep. 2017 Apr 19;7:946. doi: 10.1038/s41598-017-01044-4 (PMC5430436; doi:10.1038/s41598-017-01044-4)
Supplement: Supplementary file 1 — Supplementary Information [file 41598_2017_1044_MOESM1_ESM.pdf]

Supplementary Information

to the article entitled

[^{18}F]tetrafluoroborate-PET/CT enables sensitive tumor and metastasis *in vivo* imaging in a sodium iodide symporter-expressing tumor model.

by

S. Diocou, A. Volpe, M. Jauregui-Osoro, M. Boudjemeline, K. Chuamsaamarkkee, F. Man,

P. J. Blower, T. Ng, G. E. D. Mullen* and G. O. Fruhwirth*

***Correspondence to**

Dr. G. O. Fruhwirth and Dr. G. E. D. Mullen, King's College London, Division of Imaging Sciences and Biomedical Engineering, 4th Floor Lambeth Wing, St Thomas' Hospital, London SE1 7EH, UK. phone: 0044 207 188 8370;

e-mail: gilbert.fruhwirth@kcl.ac.uk, greg.mullen@kcl.ac.uk.

Contents

1. Supplementary Methods
2. Supplementary Figures
3. Supplementary References

1. Supplementary Methods

Cell culture

All MTLN3E-derived cell lines were cultured in alpha-MEM supplemented with 5% (v/v) fetal bovine serum (BioSera, Uckfield East, UK; FBS), L-glutamine (25 mM), penicillin (100 IU/mL) and streptomycin (50 µg/mL). FRTL -5 cells were grown in Ham's F12K medium (ATCC via LGC Standards, Teddington, UK) supplemented with 5% (v/v) FBS, L-glutamine (2 mM), insulin (10 µg/mL), hydrocortisone (10 nM), transferrin (5 µg/mL), Gly-L-His-L-Lys acetate (10 ng/mL), somatostatin (10 ng/mL), and thyroid stimulating hormone (TSH; 10 mU/mL). All cells were incubated at 37°C in a humidified atmosphere containing 5% CO_2 (v/v). All cell lines were regularly (≤3 month intervals) tested for mycoplasma (LookOut Mycoplasma PCR Detection Kit, Sigma) and found to be negative throughout the study.

[^{18}F]BF_4^- production

[^{18}F]fluoride was obtained directly from proton-irradiated [^{18}O]water (97 atom%, Isochem Ltd., Hook, UK; 11 MeV protons from a CTI RDS 112 cyclotron, beam current 30 µA, irradiation time 10–20 min). Radiolabeling was performed using an Eckert and Ziegler Modular-Lab module (Imaging Equipment Ltd., Bristol, UK). [^{18}F]fluoride (12–18 GBq) was trapped by passage of the irradiated water (4 mL) through a QMA (Waters UK, Elstree, UK) cartridge conditioned with 1.0 M sodium hydrogen carbonate. Hydrochloric acid (1.5 M, 1.2 mL) was then passed through eluting the [^{18}F]fluoride into the reactor which contained sodium tetrafluoroborate (1 mg in 0.1 mL 1.5 M HCl). The reaction mixture (1.3 mL) was heated to 80°C for 20 min, cooled to 20°C and passed through a silver cartridge (OnGuard II AG, Dionex, Leeds, UK, conditioned with 10 mL water and 10 mL air) to remove chloride and raise the pH, and then through two alumina columns (Waters SepPak Light Alumina N, conditioned with 10 mL water and 10 mL air, to remove unreacted [^{18}F] fluoride) and a sterile Millex-GS 0.22 µm filter unit (Millipore UK, Watford, UK) into a nitrogen-filled sterile vial. The process, including irradiation, synthesis and purification, took less than 1 h.

Determination of the minimum number of NIS-positive cells detectable with the

nanoPET equipment

To determine the sensitivity of the nanoPET/CT scanner (Mediso, Budapest, Hungary) in regard of our cellular model, we prepared cell pellets consisting of NIS-positive and NIS-negative cells. This assay was performed as such, because the signal levels of the NIS-positive cells per volume are dependent on their distribution within that volume; for instance, a higher signal per volume is to be expected from 1000 NIS-positive cells if they are in a pellet of their own as compared to 1000 NIS-positive cells if they are 'diluted' in a larger volume of NIS-negative cells. Therefore, we varied the amount of NIS-positive cells while the total cell number was kept constant at $5{\cdot}10^5$ cells. We chose this pellet size as it was comparable to the sizes of the small inguinal and axillary lymph nodes in healthy SCID/Beige mice, which were the primary targets of metastasis in this xenograft model.

Briefly, NIS-positive and NIS-negative cells were mixed, transferred into Eppendorf tubes and centrifuged (250 g, 5 min). Supernatants were removed and cell pellets were washed twice with HBSS before the cell pellets were subjected to 50 kBq/mL $[^{18}F]BF_4^-$ and incubated for 30 min at 37°C. Subsequently, the cells were centrifuged and washed three times with ice-cold HBSS before being pelleted at the tube bottom and imaged in the nanoPET/CT instrument for 30min. Images were analysed using the Tera-Tomo™ software package. In line with standard analytical procedures, we defined the limit of detection (LOD) to be 3-times the standard deviation above background (*i.e.* $5{\cdot}10^5$ NIS-negative cells).

2. Supplementary Figures

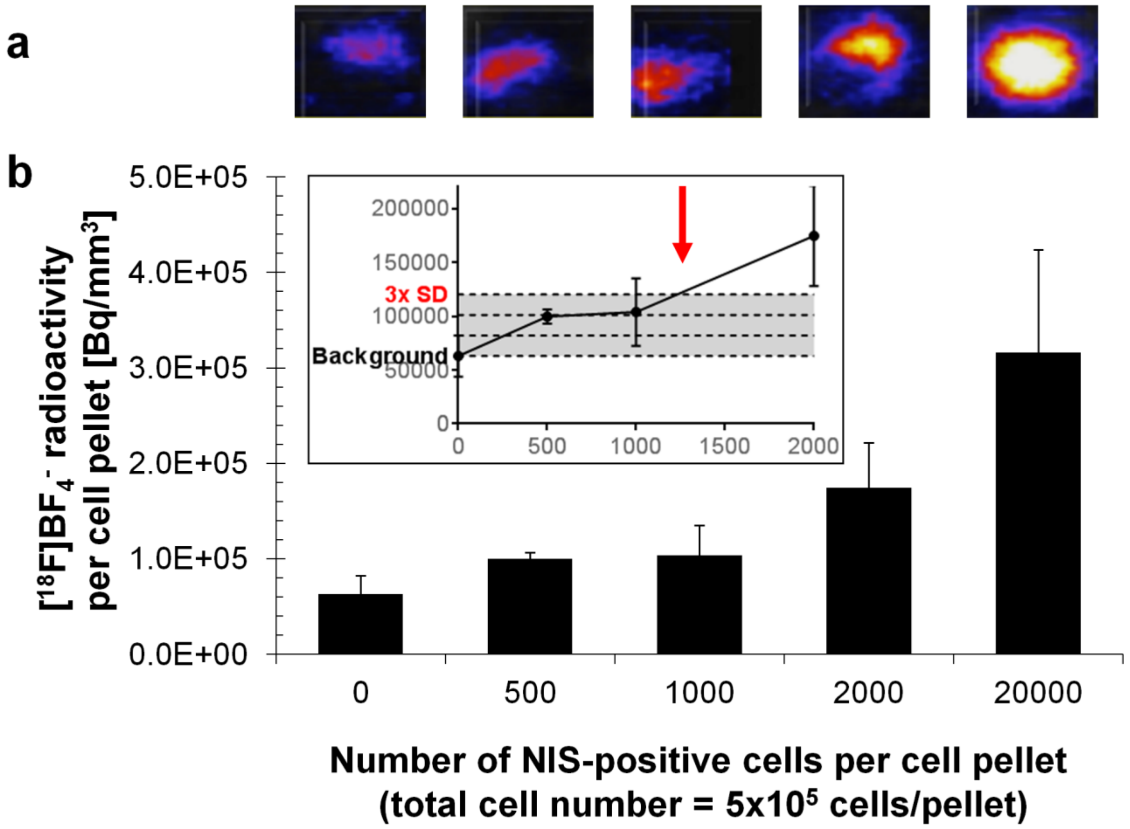

Supplementary Figure S1 | Determination of the detection limit of NIS-positive cells within a cell pellet of NIS-negative cell for the NIS radio tracer [^{18}F]BF_4^- using the nanoPET/CT equipment.

Cell pellets consisting of various amounts of NIS-positive cells within NIS-negative cells were subjected to [^{18}F]BF_4^- uptake and embedded in agarose to form a phantom for nanoPET/CT measurements (for details see Supplementary Materials and Methods). **(A)** Typical results of nanoPET/CT imaging of such phantoms are shown. **(B)** Subsequent to imaging the measured radioactivity per volume for each individual cell pellet was quantified. From triplicate experiments, we determined the limit of detection to be ~1250 NIS-positive cells (inset, red arrow) and rounded this value to our closest safely detectable cell pellet, which contained 2000 NIS-positive cells within a volume of $5{\cdot}10^5$ cells. Error bars represent standard deviations of at least three experiments.

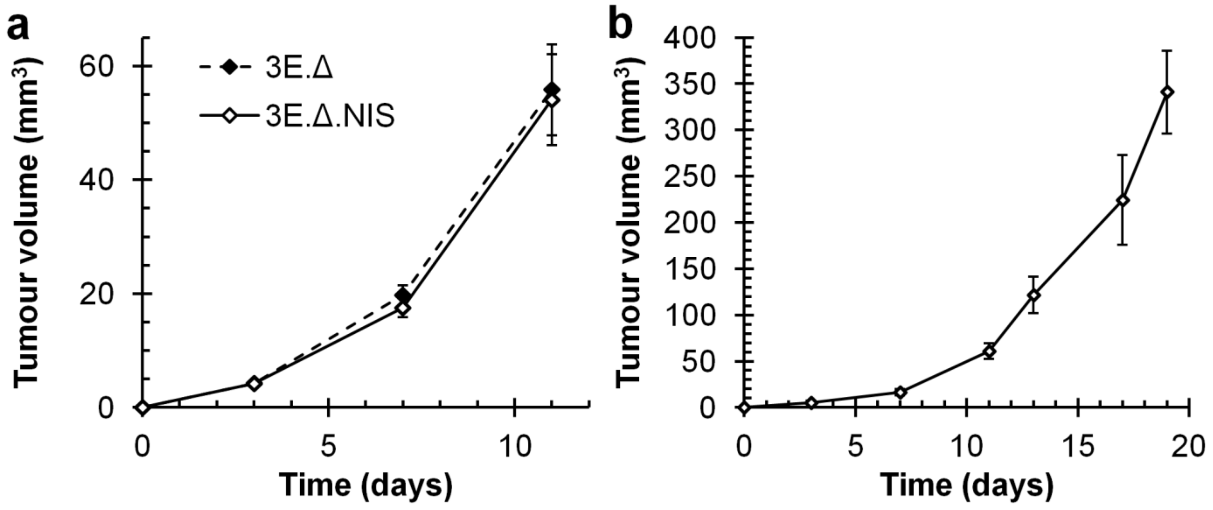

Supplementary Figure S2 | Tumor growth curves for all tumor models in this study.

All xenograft tumors in this study were monitored by calliper measurements (see Materials and Methods). **(A)** Xenograft tumor growth curves demonstrating that the reporter gene NIS-TagRFP does not have any significant impact on tumor growth rates. Tumors shown are for experiments in Fig.2 of the main manuscript. **(B)** Cumulative xenograft tumor growth curves for all other 3E.Δ.NIS tumors in our study. All error bars represent SD.

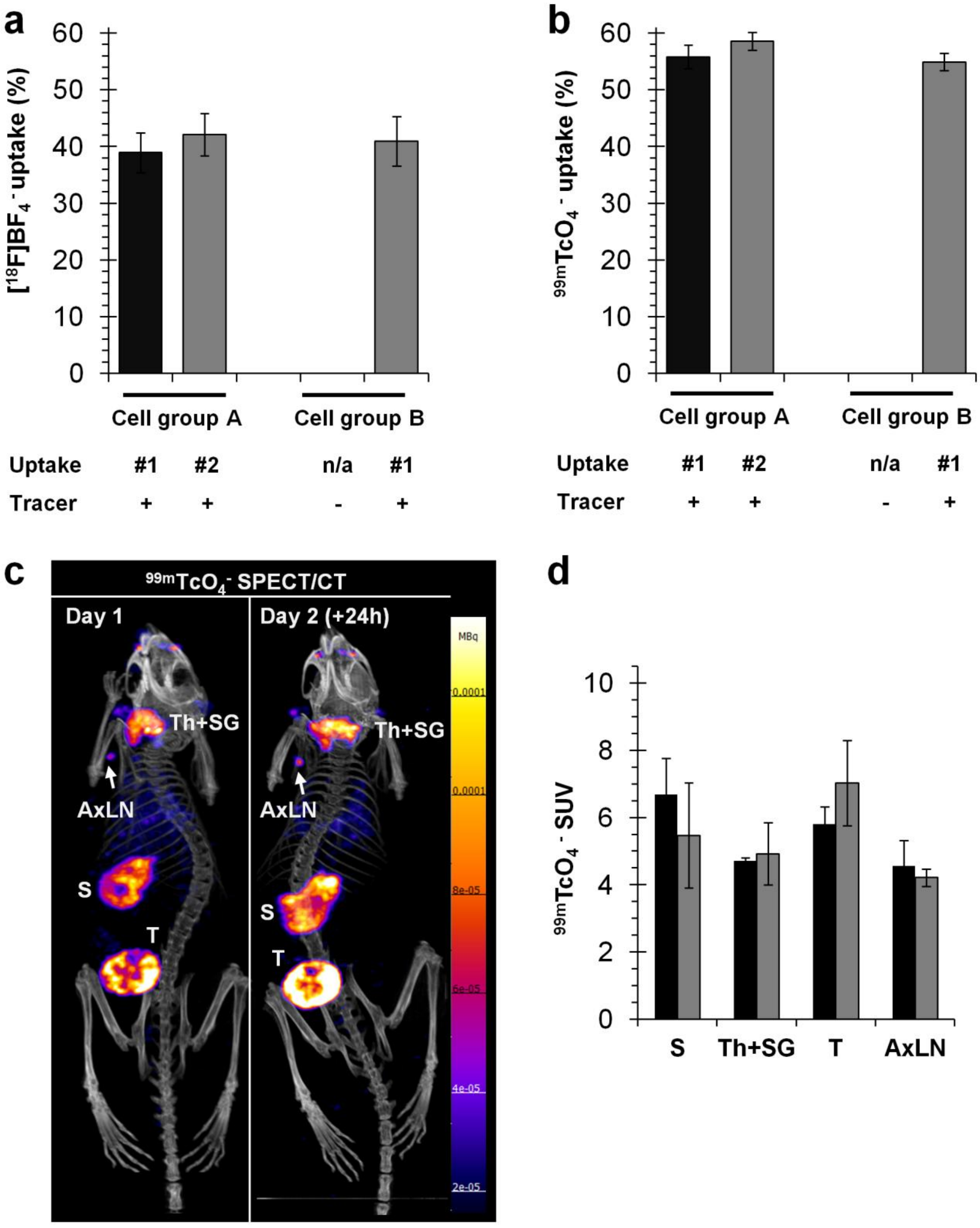

Supplementary Figure S3 | Consecutive radiotracer uptake demonstrates the validity of repeat imaging with NIS radiotracers for time intervals of 24h and above.

In vitro repeat uptake experiments with **(A)** [^{18}F]BF_4^- (a radiotracer that ceases to serve as a NIS substrate upon ^{18}F decay) and **(B)** $^{99m}TcO_4^-$ (a radiotracer that retains NIS substrate properties upon decay as ^{99m}Tc decays to ^{99}Tc) were performed with 3E.Δ-NIS cells. Cell groups were subjected to individual radiotracer uptake experiments as described in Materials

and Methods with the difference that after the uptake experiment the cells were re-plated and cultured until 24h later, when a second uptake experiment was performed with the same cells and fresh radiotracer (cell group A). Control experiments were performed like those described above, but in which cells were not receiving radiotracer but only vehicle for the first uptake round (cell group B). Data clearly demonstrate no significant differences between the first and the second uptake and no differences between equally treated cells irrespective of whether they had received radiotracer (cell group A) or vehicle (cell group B) during the first uptake experiment. **(C)** *In vivo* repeat imaging of 3E.Δ-NIS tumor-bearing animals with $^{99m}TcO_4^-$ SPECT/CT. Endogenous NIS expressing organs such as the thyroid and salivary glands (Th+SG) and the stomach (S) and cancerous tissues expressing exogenous NIS such as the primary tumor (T) and a metastatic site, namely an axillary lymph node (AxLN, arrow) are indicated. This data demonstrates the validity of the repeat imaging approach as consecutive imaging sessions are not affected by the previous one if they are at least 24h apart. **(D)** Image-based quantification from $^{99m}TcO_4^-$ SPECT imaging sessions 24h apart (three animals including the one shown in (C)) demonstrates that similar SUV values were obtained in consecutive imaging sessions (differences are not significant; P>0.05 for all comparisons between the two imaging sessions). Notably, data in (C) and (D) were obtained with the longer half life NIS radiotracer $^{99m}TcO_4^-$ (3.23-times longer half life than ^{18}F) that, in contrast to $[^{18}F]BF_4^-$, also retains its NIS substrate properties upon decay.

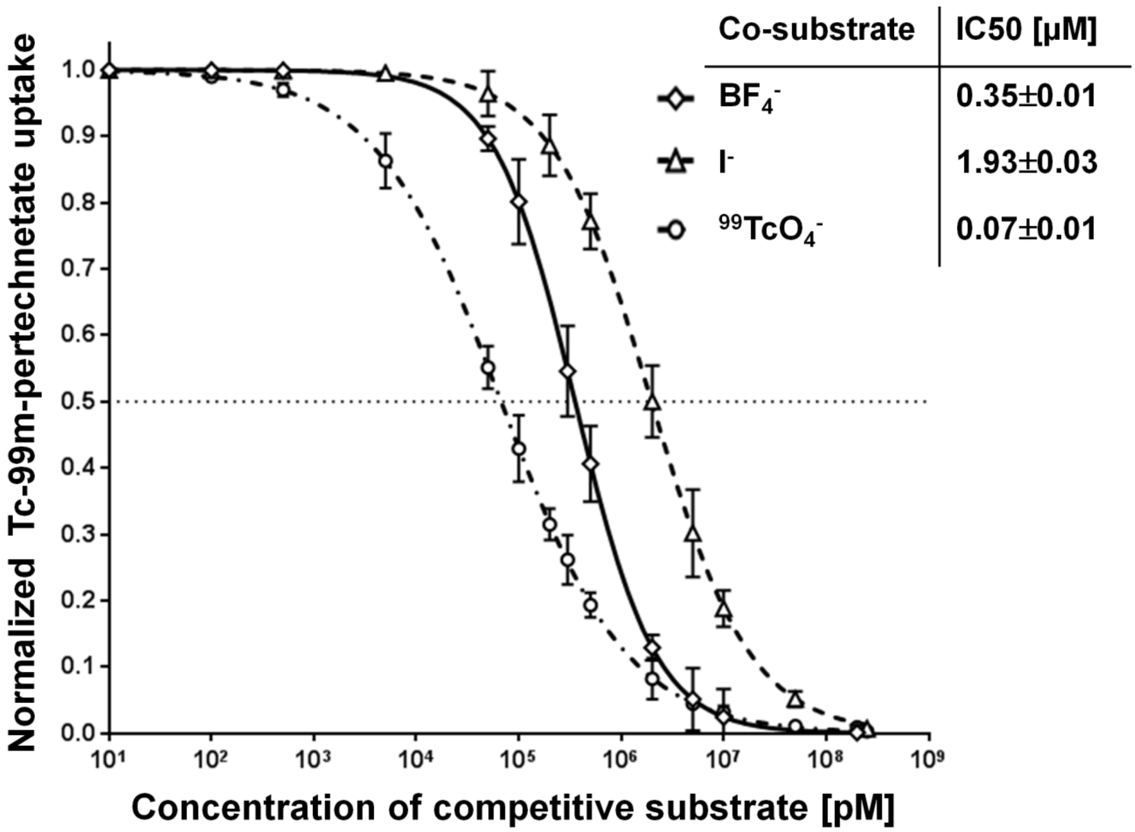

Supplementary Figure S4 | Competition assay to compare the relative IC_{50} of tetrafluorborate, iodide and [^{99}Tc]pertechnetate in the same cell line, probed with [^{99m}Tc]pertechnetate.

Uptake assays were performed as described in Materials and Methods in the main manuscript. They differed from standard uptake assays in that they were also performed in the presence of differing amounts of either cold tetrafluoroborate, cold iodide or [^{99}Tc]pertechnetate. $^{99m}TcO_4^-$ was always used at 50kBq per cell sample (consisting of 1 million cells). $^{99m}TcO_4^-$ was obtained from a ^{99}Mo/^{99m}Tc generator. However, the total pertechnetate concentration in this assay, against which the other tracers are measured in a competitive manner, requires a more detailed consideration. ^{99m}Tc has a half live of 6.02h and its theoretical specific activity is 19,260 GBq/µmol [Supplementary Reference A]. Our generator was eluted with physiological saline to provide $^{99m}TcO_4^-$ and also the unwanted $^{99}TcO_4^-$ in a solution as sodium pertechnetate. The presence of ^{99}Tc ('carrier Tc') is due to the decay of ^{99m}Tc, but also because 13.95% of ^{99}Mo decays directly to ^{99}Tc. Thus, $^{99m}TcO_4^-$ is never carrier free and the specific activity decreases rapidly after generator elution [Supplementary Reference B]. Therefore, the

mole fraction of ^{99m}Tc (MF_{Tc99m}), which contributes to the activity, is the fraction of the technetium atoms, which are ^{99m}Tc. As there were 24h between routine generator elutions, the MF_{Tc99m} was 0.277 [Supplementary Reference C]. In our assays, we used the pertechnetate always 4.5h after elution (*i.e.* after ~3/4 of its half life), which was the time point when the preparation of the 50 kBq/mL working solutions took place; including decay correction, the MF_{Tc99m} was 0.165. While theoretically the quantity of $^{99m}TcO_4^-$ in a 50 kBq/mL solution would have been 2.6 fmol, the above considerations revealed that the total amount of pertechnetate ($^{99m}TcO_4^-$ and $^{99}TcO_4^-$) was 16 fmol under the described conditions. Consequently, the working solutions for our uptake assays contained 16pM pertechnetate in total. Cell pellet radioactivity was measured by γ-counting in the ^{99m}Tc energy window (^{99}Tc did not interfere at these concentration levels due to its much weaker and different radiation). The co-substrate's effects on cellular $^{99m}TcO_4^-$ uptake were quantified and fitted to a sigmoid model using Prism software v7.01 (GraphPad, La Jolla, US). This data reveals that tetrafluorborate has a 5.6-times lower IC50 value in this pertechnetate competition assay and indicate that tetrafluorborate is a more potent substrate in these NIS-expressing cells than iodide.

3. Supplementary References

A. Eckelman WC, Volkert WA, Bonardi M. True radiotracers: are we approaching theoretical specific activity with Tc-99m and I-123. *Nucl. Med. Biol.* **35**, 523-527 (2008).

B. Ballinger JR. The influence of carrier on ^{99m}Tc radiopharmaceuticals. *J. Nucl. Med.* **46**, 224-232 (2002).

C. Lamson ML, Kirschner AS, Hotte CE, Lipsitz EL, Ice RD. Generator-produced $^{99m}TcO_4^-$: carrier free? *J. Nucl. Med.* **16**, 639-641 (1975).
